# Supplementary material for: Unveiling the Bioactive Potential of the Invasive Jellyfish Phyllorhiza punctata Through Integrative Transcriptomic and Proteomic Analyses
Source: Biomolecules. 2025 Aug 4;15(8):1121. doi: 10.3390/biom15081121 (PMC12383608; doi:10.3390/biom15081121)
Supplement: Supplementary file 1 [file biomolecules-15-01121-s001.zip › Suplementary Material/FileS3/PEPFOLD4/XP_065056435_5240/clusters.html]

 PEP-FOLD clusters


Download archive of all models  here   

|  |  |  |
| --- | --- | --- |
| # | file | sOPEP |
| # cluster 1 - Size: 11 |  |  |
|  | XP\_065056435\_5240-model1 | -10.0584 |
|  | XP\_065056435\_5240-model1.1 | -9.94136 |
|  | XP\_065056435\_5240-model1.2 | -9.94136 |
|  | XP\_065056435\_5240-model1.3 | -9.94136 |
|  | XP\_065056435\_5240-model1.4 | -9.94136 |
|  | XP\_065056435\_5240-model1.5 | -9.94136 |
|  | XP\_065056435\_5240-model1.6 | -9.94136 |
|  | XP\_065056435\_5240-model1.7 | -9.94136 |
|  | XP\_065056435\_5240-model1.8 | -9.94136 |
|  | XP\_065056435\_5240-model1.9 | -9.94136 |
|  | XP\_065056435\_5240-model1.10 | -9.94136 |
| # cluster 2 - Size: 55 |  |  |
|  | XP\_065056435\_5240-model2 | -10.0305 |
|  | XP\_065056435\_5240-model2.1 | -10.0305 |
|  | XP\_065056435\_5240-model2.2 | -10.0305 |
|  | XP\_065056435\_5240-model2.3 | -10.0305 |
|  | XP\_065056435\_5240-model2.4 | -10.0305 |
|  | XP\_065056435\_5240-model2.5 | -10.0305 |
|  | XP\_065056435\_5240-model2.6 | -10.0305 |
|  | XP\_065056435\_5240-model2.7 | -10.0305 |
|  | XP\_065056435\_5240-model2.8 | -10.0305 |
|  | XP\_065056435\_5240-model2.9 | -10.0305 |
|  | XP\_065056435\_5240-model2.10 | -10.0305 |
|  | XP\_065056435\_5240-model2.11 | -10.0305 |
|  | XP\_065056435\_5240-model2.12 | -10.0305 |
|  | XP\_065056435\_5240-model2.13 | -10.0305 |
|  | XP\_065056435\_5240-model2.14 | -10.0305 |
|  | XP\_065056435\_5240-model2.15 | -9.90884 |
|  | XP\_065056435\_5240-model2.16 | -9.90884 |
|  | XP\_065056435\_5240-model2.17 | -9.90884 |
|  | XP\_065056435\_5240-model2.18 | -9.90884 |
|  | XP\_065056435\_5240-model2.19 | -9.90884 |
|  | XP\_065056435\_5240-model2.20 | -9.90884 |
|  | XP\_065056435\_5240-model2.21 | -9.90884 |
|  | XP\_065056435\_5240-model2.22 | -9.90884 |
|  | XP\_065056435\_5240-model2.23 | -9.90884 |
|  | XP\_065056435\_5240-model2.24 | -9.90884 |
|  | XP\_065056435\_5240-model2.25 | -9.90884 |
|  | XP\_065056435\_5240-model2.26 | -9.90884 |
|  | XP\_065056435\_5240-model2.27 | -9.90884 |
|  | XP\_065056435\_5240-model2.28 | -9.90884 |
|  | XP\_065056435\_5240-model2.29 | -9.90884 |
|  | XP\_065056435\_5240-model2.30 | -9.90884 |
|  | XP\_065056435\_5240-model2.31 | -9.90884 |
|  | XP\_065056435\_5240-model2.32 | -9.90884 |
|  | XP\_065056435\_5240-model2.33 | -9.90884 |
|  | XP\_065056435\_5240-model2.34 | -9.90884 |
|  | XP\_065056435\_5240-model2.35 | -9.90884 |
|  | XP\_065056435\_5240-model2.36 | -9.90884 |
|  | XP\_065056435\_5240-model2.37 | -9.90884 |
|  | XP\_065056435\_5240-model2.38 | -9.90884 |
|  | XP\_065056435\_5240-model2.39 | -9.90884 |
|  | XP\_065056435\_5240-model2.40 | -9.90884 |
|  | XP\_065056435\_5240-model2.41 | -9.90884 |
|  | XP\_065056435\_5240-model2.42 | -9.90681 |
|  | XP\_065056435\_5240-model2.43 | -9.90681 |
|  | XP\_065056435\_5240-model2.44 | -9.90681 |
|  | XP\_065056435\_5240-model2.45 | -9.90681 |
|  | XP\_065056435\_5240-model2.46 | -9.90681 |
|  | XP\_065056435\_5240-model2.47 | -9.90681 |
|  | XP\_065056435\_5240-model2.48 | -9.89115 |
|  | XP\_065056435\_5240-model2.49 | -9.89115 |
|  | XP\_065056435\_5240-model2.50 | -9.89115 |
|  | XP\_065056435\_5240-model2.51 | -9.89115 |
|  | XP\_065056435\_5240-model2.52 | -9.89115 |
|  | XP\_065056435\_5240-model2.53 | -9.89115 |
|  | XP\_065056435\_5240-model2.54 | -9.87003 |
| # cluster 3 - Size: 6 |  |  |
|  | XP\_065056435\_5240-model3 | -9.97353 |
|  | XP\_065056435\_5240-model3.1 | -9.97353 |
|  | XP\_065056435\_5240-model3.2 | -9.97353 |
|  | XP\_065056435\_5240-model3.3 | -9.97353 |
|  | XP\_065056435\_5240-model3.4 | -9.97353 |
|  | XP\_065056435\_5240-model3.5 | -9.94858 |
| # cluster 4 - Size: 28 |  |  |
|  | XP\_065056435\_5240-model4 | -9.92478 |
|  | XP\_065056435\_5240-model4.1 | -9.92478 |
|  | XP\_065056435\_5240-model4.2 | -9.92478 |
|  | XP\_065056435\_5240-model4.3 | -9.92478 |
|  | XP\_065056435\_5240-model4.4 | -9.92478 |
|  | XP\_065056435\_5240-model4.5 | -9.92478 |
|  | XP\_065056435\_5240-model4.6 | -9.92478 |
|  | XP\_065056435\_5240-model4.7 | -9.92478 |
|  | XP\_065056435\_5240-model4.8 | -9.92478 |
|  | XP\_065056435\_5240-model4.9 | -9.92478 |
|  | XP\_065056435\_5240-model4.10 | -9.92478 |
|  | XP\_065056435\_5240-model4.11 | -9.92478 |
|  | XP\_065056435\_5240-model4.12 | -9.92478 |
|  | XP\_065056435\_5240-model4.13 | -9.92478 |
|  | XP\_065056435\_5240-model4.14 | -9.92478 |
|  | XP\_065056435\_5240-model4.15 | -9.92478 |
|  | XP\_065056435\_5240-model4.16 | -9.92478 |
|  | XP\_065056435\_5240-model4.17 | -9.92478 |
|  | XP\_065056435\_5240-model4.18 | -9.92478 |
|  | XP\_065056435\_5240-model4.19 | -9.92478 |
|  | XP\_065056435\_5240-model4.20 | -9.92478 |
|  | XP\_065056435\_5240-model4.21 | -9.92478 |
|  | XP\_065056435\_5240-model4.22 | -9.92478 |
|  | XP\_065056435\_5240-model4.23 | -9.92478 |
|  | XP\_065056435\_5240-model4.24 | -9.92478 |
|  | XP\_065056435\_5240-model4.25 | -9.92478 |
|  | XP\_065056435\_5240-model4.26 | -9.92478 |
|  | XP\_065056435\_5240-model4.27 | -9.82769 |
|  |  |  |
